# Dietary Probiotic *Pediococcus acidilactici* MA18/5M Improves the Growth, Feed Performance and Antioxidant Status of Penaeid Shrimp *Litopenaeus stylirostris*: A Growth-Ration-Size Approach

**DOI:** 10.3390/ani11123451

**Published:** 2021-12-03

**Authors:** Mathieu Castex, Eric Leclercq, Pierrette Lemaire, Liêt Chim

**Affiliations:** 1 LALLEMAND SAS, 19 rue des Briquetiers, 31702 Blagnac, France; eleclercq@lallemand.com; 2 IFREMER, Unité Lagons, Ecosystèmes et Aquaculture Durable en Nouvelle Calédonie (LEAD), B.P. 2059, 98846 Nouméa, New Caledonia, France; pierette.lemaire@ifremer.fr (P.L.); liet.chim@ifremer.fr (L.C.); 3 IFREMER, Laboratoire BRM/PBA, Rue de l’Ile d’Yeu, 44311 Nantes, France

**Keywords:** probiotic, *Pediococcus acidilactici*, shrimp, growth, carbohydrate, antioxidant status

## Abstract

**Simple Summary:**

Probiotics are increasingly documented to confer health and performance benefits across farmed animals. The study assessed the effect of dietary supplementation with the single-strain probiotic *Pedicococcus acidilactici* MA18/5M on the growth, nutritional indices, and metabolic status of the adult western blue shrimp, *Litopenaeus stylirostris*. The aim was to estimate its potential at optimizing the performance of the penaeid feed and shrimp farming industry. Supplementation with *P. acidilactici* MA18/5M improved the feed conversion efficiency and daily growth rate across fixed ration sizes; and decreased both the maintenance and optimal ration size for growth. This appeared linked to a better use of dietary carbohydrates as shown by a higher α-amylase activity, free-glucose and glycogen concentration in the digestive gland. Interestingly, *P. acidilactici* intake was also associated with a higher antioxidant status which may be linked to enhanced carbohydrates utilization. Using a fixed ration size approach under controlled laboratory conditions, the study documented a clear potential for *P. acidilactici* MA18/5M to enhance the growth, feed efficiency and metabolic health of adult penaeid shrimp during on-growing. These findings raise interesting prospects to optimize penaeid feed formulation and the performance of the shrimp-farming industry.

**Abstract:**

Probiotics are increasingly documented to confer health and performance benefits across farmed animals. The aim of this study was to assess the effect of a constant daily intake of the single-strain probiotic *Pedicococcus acidilactici* MA18/5M (4 × 10^8^ CFU.day^−1^.kg^−1^ shrimp) fed over fixed, restricted ration sizes (1% to 6% biomass.day^−1^) on the nutritional performance and metabolism of adult penaeid shrimp *Litopenaeus stylirostris* (initial body-weight, BWi = 10.9 ± 1.8 g). The probiotic significantly increased the relative daily growth rate (RGR) across all ration size s tested (Mean-RGR of 0.45 ± 0.08 and 0.61 ± 0.07% BWi.day^−1^ for the control and probiotic groups, respectively) and decreased the maintenance ration (Rm) and the optimal ration (Ropt) by 18.6% and 11.3%, respectively. Accordingly, the probiotic group exhibited a significantly higher gross (K1) and net (K2) feed conversion efficiency with average improvement of 35% and 30%, respectively. Enhanced nutritional performances in shrimps that were fed the probiotic *P. acidilactici* was associated with, in particular, significantly higher α-amylase specific activity (+24.8 ± 5.5% across ration sizes) and a concentration of free-glucose and glycogen in the digestive gland at fixed ration sizes of 3% and below. This suggests that the probiotic effect might reside in a better use of dietary carbohydrates. Interestingly, *P. acidilactici* intake was also associated with a statistically enhanced total antioxidant status of the digestive gland and haemolymph (+24.4 ± 7.8% and +21.9 ± 8.5%, respectively; *p* < 0.05). As supported by knowledge in other species, enhanced carbohydrate utilization as a result of *P. acidilactici* intake may fuel the pentose-phosphate pathway, generating NADPH or directly enhancing OH-radicals scavenging by free glucose, in turn resulting in a decreased level of cellular oxidative stress. In conclusion, the growth-ration method documented a clear contribution of *P. acidilactici* MA18/5M on growth and feed efficiency of on-growing *L. stylirostris* that were fed fixed restricted rations under ideal laboratory conditions. The effect of the probiotic on α-amylase activity and carbohydrate metabolism and its link to the shrimp’s antioxidant status raises interesting prospects to optimize dietary formulations and helping to sustain the biological and economic efficiency of the penaeid shrimp-farming industry.

## 1. Introduction

Aquaculture widely contributes to the availability of aquatic food for human consumption and relies on high-quality feed to ensure the health and performance of the animals and the sustainability of its industry. Research into the use of probiotics for aquatic animals has increased with the demand for environmentally friendly and sustainable aquaculture [1,2]. Probiotics were originally defined as microbial dietary supplements which bring beneficial effects to the host [3] and are commonly viewed as prophylactic supplements in human health [4,5]. Several reviews [2,6,7,8,9,10,11] detail the various developments made in the application of probiotics in aquatic species, including shrimp. In aquaculture, probiotics are usually used as biocontrol agents for preventing disease and/or increasing resistance to pathogens [12] and several possible action mechanisms have been suggested. Among these, the competitive exclusion of pathogenic bacteria [13,14,15] and enhancement of the immune and antioxidant defense systems against pathogenic micro-organisms [16,17,18,19,20,21] have been widely invoked. For instance, the probiotic strain *Pediococcus acidilactici* MA18/5M has been the subject of a number of evaluations with regard to the health of the penaeid shrimp *Litopenaeus stylirostris*, and its dietary use was shown to be beneficial against vibriosis [17,18,22].

Probiotics are also often used as growth promoting agents in farmed animals [23,24] and as a digestibility enhancer in ruminants [25]. Several studies have recently reported that probiotic bacteria are good candidates for improving nutrient digestion and the growth of aquatic organisms [2,11]. Benefits to the host have been reported to include an improved feed utilisation and feed value by supplying beneficial dietary compounds (vitamin B12, biotin, carotenoids, amino acids), by detoxifying potentially harmful compounds in feeds and/or by an enzymatic contribution to digestion [2,26,27]. For example, Ref. [28] showed that abalone (*Haliotis midae*) that were fed a kelp diet supplemented with *Pseudoalteromonas* sp. strain C4 exhibited an increased growth rate compared to abalone fed standard kelp. They suggested that the probiotic can play an important role in the nutrition of farmed abalone in three ways: (i) pre-digestion of alginate in kelp-based feed, (ii) increased alginate lyase activity in the abalone digestive tract and (iii) utilisation of strain C4 as a protein source. Moreover, probiotics are also considered to influence digestive processes by enhancing the population of beneficial micro-organisms [29,30], microbial enzyme activity and the intestinal microbial balance [31]. However, to date, probiotic studies carried out with shrimps have mainly focused on their increased resistance to disease [12,15,22] and population growth performance [19,32,33,34,35].

The aim of the present study was to specify the effect of *P. acidilactici* on the growth and nutrition of the shrimp *L. stylirostris*. To this end, we determined the ration size for maintenance and optimal growth by means of the growth-ration method initially proposed for fish by [36] and largely applied in fish. Its main function is to determine the quantitative daily nutritional and energy requirements for maintenance and optimal growth as well as the impact of environmental factors [36,37,38,39,40,41,42,43,44]. Surprisingly, this fundamental GR-method remains seldom applied and quantitative requirements are comparatively infrequently determined for penaeid shrimps. Refs. [45,46,47] based their studies on the GR-relation to determine the effect of feeding frequency and natural productivity of the pond on growth and feed efficiency of *Penaeus merguiensis* and *L. stylirostris*. Ref. [48] used the same approach to estimate the daily quantitative protein requirements of juvenile *Litopenaeus vannamei*. However, most studies have evaluated the effect of qualitative variations of the feed nutritional profile on growth and feed conversion. Indeed, as mentioned by [48], research on the protein requirements of penaeid shrimp has largely been concerned with evaluations of optimal dietary protein level and not with the quantitative protein requirement, since *ad libitum* feeding is generally used. The use of the GR-relation and the quantification of the daily requirements obtained from it could provide useful information to maximize penaeid shrimp production [48]. Moreover, this method could be used to evaluate and optimize the use of dietary additives.

Importantly, in many studies on crustaceans, the rearing tank is generally used as the experimental unit and a simple analysis of variance is applied to the tank’s mean data. However, Ref. [49] highlighted the advantage of using individual measurements and nested designs in aquaculture experiments. In order to analyse such designs without pseudoreplication [50], a nested analysis of variance has been identified as the correct method to use [51]. Nested ANOVA maintains both between-tank and within-tank variability in the analysis, and therefore reduces the risk of drawing invalid conclusions as can occur when using a simple analysis of variance [52]. New techniques and methods are now available for measuring several variables at the individual level for fish, as mentioned by [49]. The individual tagging of the animals is one such method [53]; however, its use for individual shrimp growth measurements remains undocumented.

In this study, we combined the GR-method and individual tagging of the test specimens in order to precisely measure the key nutritional parameters (maintenance and the optimal rations, scope for growth, gross and net conversion efficiency) and accurately assess the putative effects of the probiotic on the growth and feed efficiency. Furthermore, a biochemical analysis of free glucose, glycogen, digestive enzyme activities and total antioxidant status (TAS) in the haemolymph and digestive gland was carried out to gain further insight into the probiotic effect at physiological level. To the authors knowledge, no such approach has been previously applied to assess the effect of probiotic supplementation on nutritional parameters in shrimps.

## 2. Materials and Methods

The trial was conducted at the Saint Vincent Aquaculture Research Station of IFREMER located on the west coast of New Caledonia (Latitude 21°47′ S, Longitude 165°45′ E).

### 2.1. Experimental Diets and Dietary Probiotic

The basal diet was formulated (Table 1; 43.8% protein, 10.0% lipid; 2.0% fiber, 4502 kcal.kg^−1^ gross energy) and processed internally as follows: The ingredients were grinded using a laboratory grinder with a 1 mm screen and the meal was mixed with oil and water (30%) using a horizontal mixer. The mixture was then extruded using a meat grinder through a 3 mm die, the feed strands were dried (60 °C, 24 h) in a drying-oven to a residual moisture of below 10% and then broken-up into pellets from 4 to 5 mm in length.

The commercial probiotic tested in this study (Bactocell PA 10; Lallemand S.A.S, Blagnac, France) consisted of live, freeze-dried *Pediococcus acidilactici* MA 18/5M (Institut Pasteur, Paris, France) formulated in a powder form to a concentration of 10^10^ CFU.g^−1^ of product. The probiotic was top-coated on the pellets using 3% of fish oil as a carrier. Probiotic concentrations in each diet were reviewed after feed production against the target concentrations (Table 2) through homogenisation of the feed in peptone water, serial dilution, plating of the selected dilutions on the lactobacilli-specific de Man, Rogosa & Sharpe (MRS) plates and counting the number of Colony Forming Units after incubation (37 °C; 48 h). The control diet was also top-coated with 3% fish oil and checked for possible contamination by the probiotic strain. All feeds were stored in sealed 5-litre boxes at room temperature (~20 °C) until use.

### 2.2. Animals, Tagging and System

Locally sourced *L. stylirostris* (20-day post-larvae, PL20) were stocked at a density of 20 post-larvae per m^2^ within a single earthen pond (1000 m^2^), and reared under standard semi-intensive practices in New Caledonia for 4-month until they reached the desired size for the trial. In brief, shrimp were fed twice daily with a commercial formulated feed; the feeding rate was adjusted weekly according to the estimated mean body-weight (BW) and the amount of remaining feed in the feeding trays was assessed two hours after feeding.

Experimental shrimps were caught from an earthen pond using a cast net, transported in 50 L plastic containers filled with seawater, randomly stocked into the experimental tanks (6 shrimps/tank) and were permitted to acclimate for 1-week to the experimental conditions. Following acclimation, shrimps were individually colour tagged (NMT Elastomer System, Norwest Marine Technology, Shaw Island, WA, USA) by sub-cuticle injection in the last segment of the abdomen, just above the telson. Within each tank, it was possible to distinguish each specimen using 5 distinct tag colours and one specimen was left untagged but handled and sham-injected accordingly. Following tagging and BW measurement, animals were returned to their original tanks, at which point the trial started.

The trial was carried out in 30 self-cleaning circular polyester tanks (0.92 m^2^ bottom surface area; 536 L capacity) that were continuously supplied with natural seawater (100% renewal daily) pumped-ashore the adjacent lagoon, sand-filtered and stored in an elevated earthen reservoir for gravity supply. The water renewal rate was set at 400%.day^−1^.tank^−1^ and aeration was provided to each tank. The temperature was measured continuously using an automatic recording probe (Optic StowAway^®^ Temp; Onset, MA, USA). Water quality parameters over the trial’s duration were DO > 80%; temperature 27.2 ± 1.5 °C; salinity = 35.0 ± 0.1 ppt.

### 2.3. Experimental Design, Feeding and Sampling

The 27-day experiment was conducted using 180 sub-adult *L. stylirostris* at an initial BW (BWi) of 10.93 ± 1.78 g. The experiment tested two diet conditions (probiotic vs. control) with five fixed ration sizes (1%, 2%, 3%, 4% and 6% BMi.day^−1^; where BMi is initial biomass) per diet with three of the tanks randomly assigned to each treatment (30 tanks, 6 shrimp.tank^−1^). The set-feed rations were determined by prior experimentation, which had shown that they were completely consumed under our experimental conditions, with apparent satiation circa 7% BM.day^−1^. The probiotic treatment targeted, based on prior knowledge [17,22], a daily *P. acidilactici* 18/5M intake of 4 × 10^8^ CFU.kg^−1^ shrimp across ration sizes such that the probiotic incorporation within each diet group was adjusted to the five pre-determined ration size (Table 2). The daily pre-weighed ration per tank was delivered in four equally sized meals distributed at 7.00 a.m., 1.00 p.m., 7.00 p.m. and 1.00 a.m. using automatic feeders.

Individually tagged specimen were each measured for BW (±0.01 g) after carefully drying on soft paper at the start (immediately after colour tagging) and at the end of the trial (on the 28th day at 8.00 a.m., seven hours after the last feeding). At the end of the trial, four shrimps per tank were further sacrificed and immediately sampled for haemolymph and the digestive gland. Only shrimps in intermoult (stage C-D0) were used as digestive enzyme activity and other physiological parameters change during the moulting stage. To do so, 200 µL haemolymph was withdrawn from the ventral sinus cavity using a 1 mL sterile syringe, fitted with a 23-gauge needle. Haemolymph samples were immediately diluted in a pre-cooled saline-sodium citrate buffer (SCC; 30 mM trisodium citrate, 0.34 M sodium chloride, 1 mM EDTA) and snap-frozen in liquid nitrogen prior to storage at −80 °C until analysis. Digestive glands were removed, snap-frozen in liquid nitrogen and stored at −80 °C until analysis.

### 2.4. Analytical Protocols

#### 2.4.1. Samples Preparation

The diluted haemolymph samples were thawed under refrigeration, vortexed and assayed for glucose and Total Antioxidant Status (TAS). The digestive glands were thawed under refrigeration, divided into two parts, each of which was weighed. One part was homogenised using an ultra-turrax^®^ in a 10 mM Tris buffer (1 mM DTPA, 1 mM PMSF, pH 7.4) for protein, glucose, glycogen, α-amylase and trypsin activity assays and the other part was homogenised in an SCC buffer for TAS determination. Prior to analysis, the digestive gland homogenates were centrifuged (4000 rpm, 10 min, 4 °C) and the supernatant from two shrimps from the same tank were pooled iso-volumetrically.

#### 2.4.2. Biochemical Analysis

Total soluble proteins were determined in accordance with [54] with bovine serum albumin (BSA) standard. Glucose levels were determined using a commercial kit (Glucose RTU; bioMérieux, Craponne, France) based on the enzymatic conversion of glucose into quinoneimine and its colorimetric quantification at 505 nm. The assay was adapted to microplate manipulations following the manufacturer’s recommendation. Glycogen was extracted in the presence of sulphuric acid and phenol [55] as follows: Samples were first homogenised in trichloro-acetic acid (TCA 5%; 2 min, 16,000 rpm) then centrifuged (5 min, 3000 rpm). This procedure was carried out twice, the supernatants were then pooled, vortexed and 500 µL of supernatant was pipetted into a tube and mixed with five volumes of 95% ethanol. The tubes were then left to precipitate in an oven (37 °C, 3 h) and centrifuged (3000 rpm, 15 min). The glycogen pellet was dissolved through the addition of boiling water and concentrated sulphuric acid and phenol. The extract was then transferred into a microplate reader (four replicate per sample) and read at 490 nm.

The α-amylase activity was assayed by the Bernfeld’s method [56] using 1% soluble starch in phosphate buffer (20 mM; pH 7) as the substrate, 37 °C incubation, and an indirect measurement of the maltose released by DNS (3,5-dinitrosalicylic acid) colorimetry at 570 nm. One unit of enzymatic activity is defined as 1 mg of maltose liberated per min at 37 °C and expressed as total activity (U.mg_organ_^−1^). Trypsin was assayed by its amidase activity using benzoyl-Arginine-p-nitroanalide (BAPNA) as the substrate, following the method of [57,58]. Assays were initiated by the addition of sample supernatant, and the release of p-nitroanalide was measured at 410 nm over 15 min. A positive control of 3 mg.mL^−1^ trypsin (SIGMA) was used. One activity unit was expressed as 1 μmol of p-nitroanilide released.min^−1^.

Total Antioxidant Status (TAS) was determined using a commercial colorimetric kit (Randox TAS Assay; Randox Co., Antrim, UK). The tests quantify the total amount of antioxidants in blood by inhibiting the transformation of 2,2-azino-di-[3-ethylbenzthiazoline sulfonate] (ABTS^®^) into the radical cation (ABTS^®•+^) in the presence of a peroxidase (metmyoglobin) and H_2_O_2_ with an absorbance reading at 600 nm.

### 2.5. Calculations: Growth and Nutritional Parameters

#### 2.5.1. Relative Daily Growth Rate (RGR), K1 and K2

The relative daily growth rate (RGR) was expressed as a percentage of BWi and calculated as RGR_i_ = 100 × ((BWf − BWi)/(d × BWi)), where BWi and BWf are the initial and final body-weight, respectively, and d is the number of days between measurements. For each modality (treatment and ration size), RGR was first determined at an individual level (tagged specimens) and then at tank levels based on individual’s RGR, thereby removing any mortality from the dataset and providing a more robust estimation of RGR.

The gross feed conversion efficiency (K1) expresses the capacity to convert feed into body tissues. The net feed conversion efficiency (K2) provides a measure of the capacity to convert the amount of food available for growth, which is equal to the amount of feed consumed in excess of the maintenance ration (Rm). Both K1 and K2 data were determined for each tank according to [39] as K1 = (RGR/R) × 100 and K2 = (RGR/(R − Rm)) × 100; where RGR is the mean relative daily growth rate per tank, R the ration ingested per tank and Rm is the maintenance ration per tank, all expressed in (% BM).

#### 2.5.2. Growth-Ration (GR) and K1-Ration (KR) Curve Models

The experimental design did not allow for quantifying the shrimps’ individual feed consumption. Thus, we cannot obtain nutritional parameters at the individual level. However, the overall consumption of the ration provides a measurement of the feed consumption of the shrimps at the tank level. Then, in order to determine the correct parameters to follow, we considered a growth–ration model according to RGR means per tank. For each dietary treatment (control and probiotic diet), the relationship between RGR and ration size was analysed with a non-linear regression and GR curves were plotted. The regressions were calculated to fit the data within the range of the ration sizes (1% to 6%). The model describing the response was: (1) GR: y = y_0_ + a(1 − b^x^), where y is the tank average RGR, x is the ration ingested, and a, b are constants determined by the regression. Furthermore, values of K1 in relation to ration size were plotted (KR curve) by using the predicted values from the growth–ration model: (2) KR: y/x = (y_0_ + a(1 − b^x^))/x.

#### 2.5.3. Determination of Maintenance (Rm) and Optimal (Ropt) Rations

From the GR and KR curves, the maintenance (Rm) and optimal (Ropt) rations were determined according to [36]. Rm is the feed intake that maintains the animal without any change in its BW, and Ropt represents the feed intake that produces the greatest increase in BW for the least feed intake, in other words, it determines optimal growth. Daily Rm calculated from equation (1), when y is null, corresponds to the ration for which the RGR is null. Ropt is identifiable on the GR curve as the ration for which the tangent crosses the origin; Ropt was determined as the ration for which the KR curve reaches its maximum, and it is equal to the value of x for which the first order derivative of equation (2) is null (i.e., when dK/dR = 0).

#### 2.5.4. Scope for Growth (SFG)

The scope for growth (SFG) was defined by [59] as “the difference between the energy of the food an animal consumes and all other energy utilisations and losses”. Ref. [60] was able to demonstrate that the difference between any rations ingested, allowing growth and the maintenance ration (Rm), gave a simple measurement of SFG. Indeed, only the part of the feed allocation that is in excess of the Rm will be available for use in growth. In this study we calculated the SFG as the difference between the digestible energy (DE) fraction of the optimal ration (Ropt × DE) and the digestible energy fraction of the maintenance ration (Rm × DE) according to the following equation: SFG = DE × (Ropt − Rm).

### 2.6. Statistics

Statistical analyses were conducted using R software [61]. Prior to analysis, all data were systematically checked for normal distribution and variance homogeneity. The percentage survival rates were normalized using an arcsine transformation before analysis. Data on survival rates were tested using a one-way analysis of variance followed by a Student’s multiple comparison t-test to determine differences among ration sizes and treatments. The effects of treatments and ration size on the physiological parameters studied were assessed by a two-way analysis of variances followed by a pairwise comparisons using Fisher’s Protected Least Significant Difference (PLSD). For each parameter, four samples per tank were assayed. The effect of dietary treatment, ration size and their interaction on BWf and RGR were tested using individual shrimp data by a two-way nested analysis of variance in order to take into account a possible significant random tank effect. Indeed, variations between tanks can represent a random nuisance factor that can lead to invalid conclusions if simple ANOVAs are used [52]. With the analysis of variance model being mixed, the ration size and dietary treatment effects were tested from a test of hypothesis using the random effect (tank effect) as error term, and individual BWi was also used as a covariate in the model. The effect of dietary treatment, ration size and their interaction on K1 and K2 were tested by a two-way analysis of variance with a post hoc Student-Newman-Keuls test, whereby significant differences occurred. Due to limitations inherent to ANOVAs, the ration-size effect was further assessed within each separate diet by non-parametric Kruskall–Wallis and a Mann–Whitney test was used to determine differences between diet at each ration size. The GR- and KR-curves models were determined and plotted for both experimental using Sigmaplot^®^ software (SPSS Inc.). Statistically significant differences between experimental groups were reported at *p* < 0.05, if not otherwise stated. Data are given as a tank means ± standard deviation of triplicate tanks (n = 3).

## 3. Results

### 3.1. Survival, Growth and Nutritional Parameters

During the experiment, the shrimp consistently consumed all the feed provided whatever the ration size, such that the amount of feed distributed was equal to the amount of feed ingested. Zootechnical results at the end of the trial are shown in Table 3. The mean final survival rate was 81.6 ± 15.0% without a diet or ration-size effect on this parameter even at a low feeding rate. The mean BWi was 10.93 ± 1.78 g with no significant difference between dietary groups (control or probiotic diet). However, significant differences due to the random allocation of shrimps to ration size and treatment groups were detected (Table 3). Accordingly, BWi was used as a covariate to statistically assess the diet and ration-size effect on BWf. RGR was used for a posteriori analysis to compare growth according to treatment and ration size. No random tank effect was detected when individual RGR data were used with a mixed ANOVA.

**Figure 1:**
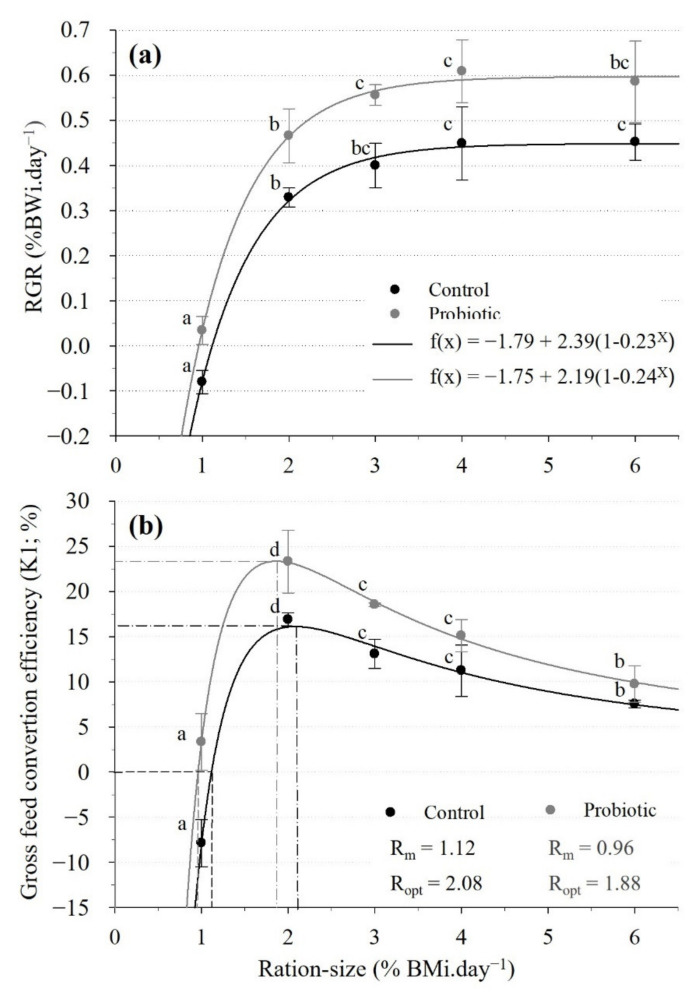
(**a**). Growth-ration (GR) curves determined per diet group from tank mean data and (**b**) Gross feed conversion-efficiency ratio (KR) curves determined per diet group from respective GR curves over the 27-day trial duration. Optimum ration size for growth (Ropt; broken lines) were calculated from the first order derivative of the KR equation (i.e., when (dRGR/x)/dT = 0). Data shown as mean ± SD, n = 3 with 4 shrimps/tank assessed. Different letters indicate significant differences between ration sizes within diets (Student-Newman-Keuls test; *p* < 0.05).

Ration size had a significant effect on RGR (Table 3 and Table 4; Figure 1a). *A posteriori* tests indicated a significant increase in RGR between 1, 2 and 3% rations for probiotic treatment and between 1, 2 and 4% rations for control (Figure 1a). No statistical difference was found between RGRs at 3%, 4% and 6% BM. The probiotic treatment resulted in a significantly higher RGR compared to the control at 1, 2, 3% (*p* < 0.01) and 4% (*p* < 0.05) but not at 6% ration size. Furthermore, a probiotic diet effect on BWf was detected when a mixed ANOVA was applied (Table 4) along with a significant random tank effect which was not detected when a classic ANOVA was applied (Table 3).

For both diets, the growth ration curves were found to fit non-linear regressions which plateaued at a ration size over 3%. The equations of the curves obtained for each treatment are indicated in Figure 1a, and adjusted R-squares were over 99% for both regressions. From these equations, Rm and Ropt were obtained by calculation. Rm and Ropt were both lower, by 16.8% and 11.3%, respectively, in the probiotic compared to control group. As the probiotic induced a parallel drop in the maintenance ration (Rm) and in the optimal ration (Ropt), the scope for growth (SFG), which is the difference between the digestible energy of the two rations, is similar for the control and probiotic group (Table 5).

Diet and ration size had a significant effect on K1 (Table 4). The KR-curves (Figure 1b), as derived from the GR-curve models, showed that, for both diets, K1 increased significantly from 1 to 2% ration size and progressively decreased thereafter with increasing ration size. The probiotic compared to the control group had a significantly higher K1 at 1, 2 and 3% but not at larger ration sizes (Table 6) and differences in K1 between diets decreased with increasing ration sizes above Ropt (Figure 1b). K1 reached maximum values of 16.2% and 23.2% at Ropt of 2.08% BM.day^−1^ and 1.88% BM.day^−1^ for the control and probiotic treatment, respectively (Figure 1b).

The K2 values were not calculated for the control group fed at the ration size of 1%, as the mean relative growth rate was negative. Additionally, K2 was found to significantly decrease with increasing ration sizes (Table 4 and Table 6) and was significantly higher at the 2% compared to a 3% ration size in both diets. The probiotic group exhibited significantly higher K2 values than the control at feeding rates of 2% and 3% BM.day^−1^. Finally, aside for K1, no significant interaction between diet and ration size were detected for any of the variables tested (Table 4).

### 3.2. Biochemical Parameters

#### 3.2.1. Digestive Enzymes

**Figure 2:**
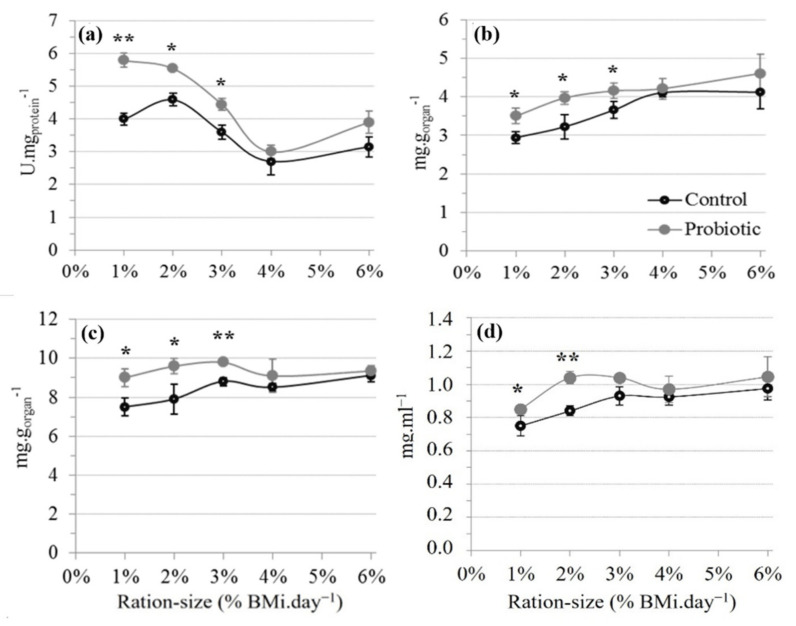
(**a**) α-amylase activity, (**b**) glycogen level and (**c**) glucose level in digestive gland; (**d**) glucose level in haemolymph of *L. stylirostris* according to ration size and dietary treatment at the trial’s end-point. Data shown as mean ± SD, n = 3 with 4 shrimps/tank assessed. Asterisk indicate significant differences between diets at each ration size (2-way ANOVA, PLSD; * *p* < 0.05, ** *p* < 0.01).

There were significant diet and ration-size effects without interactions on α-amylase specific activity in the digestive gland (Table 7). The specific activity of α-amylase decreased overall with increasing ration size in both diet groups; and significantly higher activities were measured in the probiotic compared to the control groups at ration sizes of 1%, 2% and 3% (Table 7; Figure 2a). There was a significant diet effect and diet × ration size interaction on the specific activity of trypsin in the digestive gland (Table 7). In the control group, trypsin activity decreased with increasing ration size, reaching levels twice lower when fed at 6% compared 1% BM.day^−1^ (Table 8). In comparison, trypsin activity was not affected by ration size (*p* > 0.05), instead remaining consistently high in the probiotic group. Variations in the amylase/trypsin ratio were not explained by any of the parameters tested (Table 7). Only one significant difference between treatments was found at 1% ration size, where probiotic fed shrimps showed higher values for this ratio (Table 8).

#### 3.2.2. Glucose and Glycogen Content

There were significant diet and ration-size effects on glycogen content in the digestive gland (Table 7). The probiotic group exhibited significantly higher levels compared to the control at ration sizes of 1%, 2% and 3% while no difference was detected at higher feeding rates (Figure 2b). Within diets, glycogen content statistically increased by up to 4% and up to 2% in the control and probiotic group, respectively. Free glucose in the digestive gland was also significantly higher in the probiotic compared to the control group at ration sizes of 1, 2 and 3% (Figure 2c) with levels remaining unchanged across ration sizes. In comparison, in the control group, free glucose in the digestive gland increased between ration sizes of 1% and 3%, 4%, and 6%; thereby reaching the consistently high levels measured across the probiotic group at a 4% ration size and above.

In the haemolymph, free glucose slightly, but not significantly, increased up to the ration sizes of 3% and 2% in the control and probiotic groups, respectively (Table 7; Figure 2d) and reached an apparent saturation level close to 1mg.mL^−1^ in both diet groups. Compared to the control, the probiotic group had a significantly higher haemolymph glucose concentration at a ration size of 1% and 2%.

#### 3.2.3. Total Antioxidant Status

**Figure 3:**
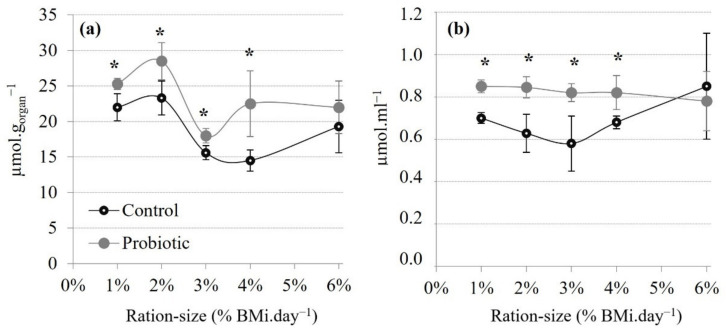
Total Antioxidant Status (TAS) in (**a**) the digestive gland and (**b**) haemolymph of shrimps from each treatment according to each ration size at the trial’s end-point. Mean ± SD, n = 3 with 4 shrimps/tank assessed. Asterisk indicate significant differences between diets at each ration size (2-way ANOVA, PLSD; * *p* < 0.05).

There were significant diet and ration-size effects on TAS of the digestive gland; and a significant ration-size effect on TAS of the haemolymph (Table 7) without interactions. In the digestive gland, TAS decreased overall with an increased ration size (Figure 3a) reaching significantly lower values at 3%, 4% and 6% compared to 1% and 2% in both diets. On the other-hand, haemolymph TAS remained consistent across ration sizes (Figure 3b). With regard to the diet effect, TAS levels in both the digestive gland and haemolymph were significantly higher in the probiotic compared to the control group across all ration sizes tested, except at 6%.

## 4. Discussion

### 4.1. GR-Curves and Nutritional Parameters in the Control Diet

The two growth-ration (GR) models established in this study for sub-adults *L. stylirostris* fed a control or probiotic diet were each based on five fixed ration sizes that were restricted and systematically fully ingested. This is of particular importance as a precise measure of feed intake is essential to the accuracy of this type of model and is particularly difficult to measure in shrimp, owing to their slow-feeding behaviour.

The GR-curves obtained from the mean RGR values per tank (Figure 1a) were used along with the KR-curves, expressing the relationship between ration size and gross feed conversion efficiency, K1 (Figure 1b), to determine the daily nutritional requirements of the shrimp for maintenance (Rm) and optimal growth (Ropt; [39]). The fundamental GR- and KR-relationships are overall well documented in fish [36,37,38,39,40] but have only rarely been investigated in shrimp [45,46,47,48]. Ref. [48] used various daily ration sizes to estimate the daily protein requirements of juvenile *L. vannamei*. The GR-relation in *L. stylirostris* is similar in appearance to those described for some fishes [36,62,63] and can be fitted to a second-order polynomial regression. However, the literature is equivocal with respect to the shape of the feeding relationship of growth in marine fish. Both linear [38,40] and non-linear (asymptotic; [37,39]) relationships are described, and this may also pertain to the range of ration sizes tested. In this study, digestible Rm of the control diet was equal to 9 g of feed.day^−1^.kg^−1^ shrimp (Table 5) which corresponds to a digestible energy (DE) of 38.14 kcal.day^−1^.kg^−1^ shrimp (159 kJ.day^−1^.kg^−1^ shrimp). This value, which is an estimate of the maintenance energy requirement, is 30% greater than that of the fasting heat production or standard metabolism determined by measuring oxygen consumption of fasting shrimp *L. stylirostris* (116 ± 7.7 kJ. day^−1^.kg^−1^ shrimp; BW = 10.6 ± 0.4 g; n = 9, 28 °C; [46]). This result is consistent because it is well accepted that the maintenance energy requirements are between 30 and 60% greater than for basal metabolism [64] and confirms the accuracy of the measurement of Rm from the GR- and KR-models for shrimp. Standard metabolism requirements are typically estimated directly, by measuring oxygen consumption rates and ammonia excretion. This approach is analytically precise but requires the confinement of the shrimp in special apparatus which may affect the animal’s response and the results are usually recorded over short time-periods (24 h to 48 h). Under such conditions, metabolic requirements may be over- or underestimated if handling stress [65,66] and moulting stage [67] are not considered. In the present study, the maintenance energy requirement and Rm were estimated from an experimental period covering several weeks, hence, fully integrating various events associated with the shrimp’s biological rhythms and cycle (moulting cycle, feeding, growth, activity and rest) as well as “real-life” fluctuations in environmental, social and zootechnical parameters.

The maximum gross conversion efficiency (K1) of sub-adult *L. stylirostris* fed the control diet was 16.88 ± 0.75% under our experimental conditions. This value is approximately half of that measured value in the same experimental conditions with younger *L. stylirostris* (7.9 ± 0.4 g, n = 150; K1 = 40%; [66]). The lower K1 in this study may reflect the weaker capacity of larger *L. stylirostris* specimens to transform feed into body tissues compared to smaller ones [66]. We also observed a gradual decline in K1 at ration sizes greater than Ropt, which may be the result of a quicker digestif transit when feed intake increases. Similarly, the net conversion efficiency (K2) of the control diet was higher at a ratio of 2% BWi.day^−1^ and then decreased at a ratio of 3% BWi.day^−1^ and more. Therefore, shrimp seem to require less feed per unit of weight-gain when the ration size is restricted as previously observed in *P. monodon* [68]. The maximum K2 obtained in our study (36.3 ± 1.9%) was within the range of values obtained for the larval stage and juveniles of other crustaceans [69,70,71].

### 4.2. Effects of the Probiotic on Growth and Nutritional Parameters

Probiotic studies in shrimps more often focus on health, increased disease resistance and related modes of action while their effect on growth remains overall less addressed. Amongst others, Refs. [32,33,72,73,74,75,76] investigated the effect of *Lactobacillus sp*., *Bacillus sp*. photosynthetic bacteria (*Rhodobacter sphaeroides*) and Gram-negative bacteria (*Enterobacter hormaechei*) on the growth of *M. rosenbergii*, *Fenneropenaeus indicus* and *L. vannamei*. In their experiments, shrimps were fed *ad libitum* and the effect of the probiotic were determined based on growth rate, feed conversion ratio and digestive enzyme activities such as protease, lipase and amylase. The authors usually linked the observed benefit of the probiotic on growth to enhanced digestion and nutrients absorption and to higher activities of digestive enzymes. In the present study, (Figure 1), we examined the effect of a constant daily intake of *P. acidilactici* on the GR and KR-relationships initially proposed by [36]. The GR-curves obtained for each dietary treatment were parallel with the probiotic curve above. Dietary probiotic intake at a dose of 4 × 10^8^ CFU.day^−1^.kg ^−1^ shrimp therefore appeared to promote growth compared to the same non-supplemented diet for a given ration size. Accordingly, based on statistical analysis, RGR was found to plateau at a lower ration size for the probiotic (3% BM.day^−1^) compared to the control (4% BM.day^−1^), suggesting that maximum growth is reached at a smaller ration size when the diet is supplemented with *P. acidilactici*. Moreover, and interestingly, shrimps fed with the probiotic diets showed reduced Rm and Ropt and increased K1 and K2. At Ropt and despite a smaller Ropt, the probiotic diet resulted in a RGR increase of over 36% compared to the control (4.5 g.kg^−1^.day^−1^ and 3.3 g.kg^−1^.day^−1^, respectively). Accordingly, K1 max was improved by 38% and K2 to Ropt by 37% in the probiotic compared to the control diet, which taken together indicate a better transformation of the feed into shrimp growth. Finally, the present data show that at a 1% ration size, shrimps fed the probiotic diet did not lose weight while control shrimps did (RGR = 0.3 g.kg^−1^.day^−1^ and -0.08 g.kg^−1^.day^−1^ for the probiotic and control groups, respectively).

Taken together, these results indicate that shrimps supplemented with probiotic require less feed to reach maintenance and optimal growth and that growth at Ropt is also superior, suggesting enhanced feed utilisation. Two other hypotheses may also explain these results. Firstly, the probiotic could provide some growth factors or essential nutrients favouring growth, as demonstrated with other probiotic strains [28]. Second, the probiotic could result in a decreased metabolic demand as suggested in this study by the lower feed requirement for maintenance and by the fact that probiotic-fed shrimps did not lose body-weight at 1% BM.day^−1^, unlike in the control. This could be evaluated by comparing oxygen consumption rate of shrimps under both dietary regimes. It must be noted that the enhanced growth and nutritional performance as a result of the probiotic intake were documented under fixed, restricted rations (below satiation), and over a short grow-out period under laboratory conditions. Further trials must therefore be performed under commercial conditions and through feed management over a longer timeframe to assess the potential contribution of the probiotic at farm-level.

### 4.3. Dietary Carbohydrates (CBH) Utilization and the Effect of Probiotic

Carbohydrates are an important energy source in shrimp diets favouring growth and fat deposition [77]. However, CBH are not efficiently utilized by shrimp which possess low carbohydrate digestion capacity and a low plasma glucose regulatory ability [78]. By applying a range of fixed and restricted rations, the study assessed the effect of a graded level of CBH intake on CBH utilization with or without probiotic supplementation.

Glycogen and free glucose values measured in this study were within the range previously reported for *L. vannamei* [79]. In the control group, the glycogen content of the digestive gland tended to increase with increasing ration size until apparent saturation above 3% BM.day^−1^ with a similar trend for free glucose level. In comparison, probiotic fed shrimps, overall, showed higher and more consistent glucose and glycogen levels. Ref. [80] reported the saturation of the glycogen level in the digestive gland for *L. stylirostris* fed over 21% dietary carbohydrates (CBH); which they suggested as the maximum dietary CBH level and apparent maximum capacity for dietary CBH utilization. However, in their studies, feed intake was not measured, making quantitative requirements for CBH impossible to determine. In an *L. vannamei* study, the same authors [81] reported the saturation curve of glycogen level in the digestive gland observed for diets with dietary CBH levels higher than 33%. In the present study, and based on glycogen levels in the digestive gland, it can be estimated that the control shrimps reached a maximum capacity to use CBH at a feed intake close to 4% BM.day^−1^, compared to a lower ration size of 2% to 3% BM.day^−1^ when supplemented with the probiotic. It can therefore be hypothesized that probiotic supplementation resulted in a more efficient use of available dietary CBH until saturation was reached. Once saturation is reached at ration sizes above 3% BM.day^−1^, the probiotic was fond to have no further discernible effects on CBH. This is supported by the absence of a significant probiotic effect on the glycogen and glucose levels of the digestive gland at these higher ration sizes, where a reduced probiotic effect on growth and gross feed efficiency was also observed.

In shrimps, once feed is ingested, starch is first processed by α-amylase to produce oligosaccharides and then glucose through α-glucosidase. This system gives rise to the slow liberation of glucose into the blood and explains why starch is viewed as an efficient CBH source in shrimp feeds [82]. Refs. [80,81] showed that in *L. stylirostris* and in *L. vannamei*, the hydrolysis of dietary starch by α-amylase could be limited by dietary CBH, and that α-glucosidase was directly related to but not limited by dietary CBH level. These authors also reported a saturation of glycogen and α-amylase specific activity above the same level of dietary CBH [80]. In this study, the higher glycogen and glucose contents of the digestive gland in probiotic-fed shrimps fed with a lower ration size (1, 2 and 3% BM.day^−1^) were associated with a higher α-amylase specific activity and haemolymph glycemia (1% and 2% BM.day^−1^). This strongly supports the hypothesis of a better utilisation of dietary CBH through *P. acidilactici* supplementation via the stimulation of α-amylase specific activity. Moreover, as for amylase, trypsin activity decreased with an increasing feeding rate which may, together, partly explain the lower feed efficiency recorded at larger ration sizes. Finally, the stable amylase/trypsin ratio measured across ration sizes in this study was consistent with values previously reported in *L. vannamei* [83] and with these authors’ suggestion that its alteration would mainly be linked to changes in dietary composition, as previously reported [83].

Several authors [33,73] have assumed that probiotics may stimulate the production of endogenous enzymes and it is possible that the probiotic produces substances, such as vitamins, which will specifically influence some digestive enzyme activities. For instance, vitamin C has been found to increase the activity of amylase [84] in shrimps, and growth factors presented in some feed ingredients have increased specific activities of amylase, trypsin and total proteases in *Marsupenaeus japonicus* [85]. Moreover, in an unpublished study, Ref. [86] was able to show that some extracellular protein products secreted by *Lactobacillus farciminis* MA27/6R and *Lactobacillus rhamnosus* MA27/6B were able to specifically enhance trypsin and α-amylase activity in *Artemia*. Besides, dietary probiotics may also exert their influence on digestive functions indirectly by modulating the composition of the endogenous intestinal microbiota [87], which has not been addressed here. This hypothesis is further supported by recent investigations regarding the functional role of the intestinal microbiota of shrimp and the link with the nutritional and phycological status of the animal [88,89].

### 4.4. A Link between Carbohydrate Metabolism and the Antioxidant Status

Ref. [90] previously documented that an increased concentration of liver-glycogen content, from increased dietary CBH intake, resulted in a decreased activity of the main antioxidant enzymes superoxide dismutase and catalase. This apparent link between CBH metabolism and the activity of antioxidant enzymes was tentatively explained by free glucose and simple sugars as a direct scavenger of OH-radicals [91,92] as well as a potent stimulant of the pentose phosphate shunt that regenerates NAD^+^ to NADPH hence, affecting the cellular redox status [93].

In a prior study, we reported decreases in superoxide dismutase and catalase activity in *L. stylirostris* fed *P. acidilactici* compared to non-supplemented shrimps [17]. In the present and subsequent study, Total Antioxidant Status (TAS) was measured to determine whether a potential probiotic effect on dietary CBH utilization would be concomitant with an altered antioxidant status. In the digestive gland, TAS decreased for greater ration sizes (3, 4 and 6% BM.day^−1^) with mean values similar to those previously reported (12.88 ± 0.63 µmol.g_organ_^−1^) in *L. stylirostris* fed *ad libitum* [18]. It has been demonstrated that feed intake and digestion increase the aerobic metabolism of *L. stylirostris* [66], while postprandial metabolism is known to rise with increased feed ingestion in isopod *Ligia pallarii* [94]. As the postprandial metabolism increases with the amount of feed consumed, the production of ROS is also expected to increase and, therefore, the TAS level to decrease as has been observed here. In crustaceans, the mobilization of antioxidant defenses may be particularly evident and critical in the lipid-storing digestive gland [95] against the risk of lipid peroxidation at greater ration sizes.

Finally, the dietary probiotic was found to significantly increase TAS level in both the haemolymph and digestive gland (except for at the highest ration size of 6%), in line with previous reports [18]. The positive effect of *P. acidilactici* on the antioxidant status of the shrimp could be the result of the better use of dietary CBH in probiotic-fed shrimp, as suggested by the work of [90] and as is overall supported by the data presented in this study. However, the mode of actions of probiotics are overall very diverse and a combination of several action mechanisms is expected to be involved for any given (generalist) probiotic [1]. For instance, the probiotic effect on antioxidant status might also be based on the antioxidant properties of the strain, as observed for other lactic acid bacteria used as probiotic [96].

## 5. Conclusions

In conclusion, the growth-ration method applied in this study was effective at documenting a clear contribution of a dietary probiotic on the growth and feed efficiency of sub-adults *L. stylirostris* fed a fixed ration under controlled conditions. The optimal ration (Ropt) was 1.88% BM.day^−1^ in the probiotic group compared to 2.08% BM.day^−1^ in the control, while RGR and Ropt were also improved in the probiotic group. The experimental approach combined individual tagging to statistically assess the effect of ration size on growth, followed by establishing the GR-relation that links ration size to the mean relative growth-rate per tank. This combined approach is proposed as a useful way of understanding how a feed additive can influence shrimp growth and feed efficiency. The effect of dietary *P. acidilactici* MA18/5M on α-amylase activity and CBH utilization and metabolism, and its link to the shrimp antioxidant status should be further studied. This study warrants further research on the contribution of this probiotic under commercial (feed) management over the grow-out cycle, as it presents the prospect of optimizing dietary formulation, as well as the biological and economic efficiency of the shrimp-farming industry.

## Figures and Tables

**Table 1:** (**a**) Raw material composition, (**b**) proximate composition and (**c**) energy content of the basal experimental diet.

(a) Ingredients (g/kg)	
LT fish meal ^(a)^	300
Soybean meal ^(b)^	200
Wheat meal ^(c)^	370
Wheat gluten	70
Fish oil	20
Soy oil	20
Soy lecithin ^(d)^	20
Shrimp Vitamin premix ^(e)^	0.5
Shrimp trace mineral premix ^(f)^	1
Stay C ^(g)^	0.4
**(b) Proximate Analysis**	
Protein ^(1)^ (%, DM basis)	43.8
Fat ^(2)^ (%, DM basis)	10
Fiber ^(3)^ (%, DM basis)	2
Ash ^(4)^ (%, DM basis)	6.9
**(c) Energy Content (kcal.kg^−1^)**	
Gross energy ^(5)^	4502
Digestible energy ^(6)^	3376

LT, low temperature; DM, dry matter. ^(a)^ Chilean low temperature fish meal from anchovy and jack mackerel; ^(b)^ Dehulled soybean meal, solvent extracted; ^(c)^ Whole wheat gran for animal feed; ^(d)^ Ultrales© lecithin (ADM lecithin, Decatur, IL, USA); ^(e)^ and ^(f)^ SICA Cie (Noumea, New Caledonia, France); ^(g)^ Vitamin C (330 mg.kg^−1^; DSM, Basel, Switzerland) ISO5983 standard; ^(1)^ ISO5983 standard; ^(2)^ NF V18-117/B standard; ^(3)^ NF V03-040 standard; ^(4)^ NF V18-101 standard; ^(5)^ Determined by calorimetric bomb (Parr^®^, USA, calibrated by benzoic acid). ^(6)^ Calculated using the concentration of chromic oxide in feed and feces (Not presented).

**Table 2:** Expected and measured probiotic concentration (*Pediococcus acidilactici*) in the probiotic diet prepared at different concentration for each ration size in order to achieve a daily probiotic intake of 4 × 10^8^ CFU.kg^−1^ shrimp. Mean ± SD. Measured count was always within the acceptable range (<0.5 log difference between expected and measured count).

Daily Ration Size (% BMi.day^−1^)	*P. acidilactici* Count (×10^7^ CFU.g^−1^ Feed)	
	Expected	Measured
1	4.00	3.8 ± 0.4
2	2.00	2.5 ± 0.2
3	1.33	1.5 ± 0.3
4	1.00	0.87 ± 0.05
6	0.67	0.65 ± 0.05

BMi, initial tank biomass.

**Table 3:** Survival, body-size and growth of *L. stylirostris* per diet group at each ration size. Mean ± SD. For each parameter, different letters within the same raw indicate significant differences between diets within each ration size (Mann–Whitney test, *p* < 0.05). Ration-size effects assessed by Kruskal–Wallis test.

Daily Ration Size (% BMi.day^−1^)	Survival (%)		BWi (g)		BWf (g)		RGR (% BWi.day^−1^)	
	Control	Probiotic	Control	Probiotic	Control	Probiotic	Control	Probiotic
1	83 ± 17 ^a^	83 ± 17 ^a^	11.55 ± 0.10 ^a^	11.02 ± 0.31 ^b^	11.25 ± 0.07	11.06 ± 0.40	−0.08 ± 0.03 ^a^	0.03 ± 0.03 ^b^
2	75 ± 12 ^a^	78 ± 10 ^a^	10.70 ± 1.42 ^a^	11.68 ± 0.44 ^a^	11.64 ± 1.54	13.47 ± 0.22	0.33 ± 0.02 ^a^	0.47 ± 0.06 ^b^
3	78 ± 25 ^a^	83 ± 17 ^a^	11.88 ± 0.59 ^a^	10.70 ± 0.31 ^b^	13.70 ± 0.60	12.15 ± 0.50	0.40 ± 0.05 ^a^	0.56 ± 0.02 ^b^
4	89 ± 19 ^a^	83 ± 29 ^a^	11.15 ± 0.41 ^a^	10.31 ± 0.69 ^a^	12.03 ± 1.55	11.81 ± 0.67	0.45 ± 0.08 ^a^	0.61 ± 0.07 ^b^
6	83 ± 0 ^a^	78 ± 10 ^a^	10.84 ± 1.19 ^a^	9.62 ± 0.43 ^a^	12.07 ± 1.01	11.06 ± 0.98	0.45 ± 0.04 ^a^	0.59 ± 0.09 ^a^
Ration-size effect	n.s.	n.s.	n.s.	**	-	-	***	***

BMi, initial tank biomass; BWi, initial body-weight; BWf, final body-weight; RGR, relative growth-rate. n.s., non-significant; ** *p* < 0.01; *** *p* < 0.001.

**Table 4:** Statistical significance (*p*-values) of diet, ration size and their interaction, and of initial body-weight and tank effects for each growth parameter determined based on type III sum of squares from factorial ANOVA. When nested ANOVA were applied, ration size and treatment effects were systematically tested from a test of hypothesis using the tank random effect as the error term. (n.s., not significant; * *p* < 0.05; ** *p* < 0.01; *** *p* < 0.001).

Performance Indices		Diet	Ration Size	BWi	Diet × Ration Size	Tank Effect
BWf ^(1)^	(g)	***	***	***	n.s.	**
RGR ^(1)^	(% BWi.day^−1^)	***	***	n.s.	n.s.	n.s.
K1 ^(2)^		***	***	n.a.	*	n.a.
K2 ^(2)^		**	***	n.a.	n.s.	n.a.

BWf, final body-weight; BWi, initial body-weight; RGR, relative growth-rate; K1, gross feed conversion efficiency; K2, net feed conversion efficiency. ^(1)^ Nested two-way analysis of variance with initial body-weight used as covariate; ^(2)^ Two-way analysis of variance; n.a. not applicable.

**Table 5:** Gross (K1) and net (K2) feed conversion efficiency by *L. stylirostris* per test diet at the ration sizes tested. Mean ± SD, n = 3. For each parameter, different letters within the same raw indicate significant differences between diets (Mann–Whitney test, *p* < 0.05). The ration size effects were assessed by a Kruskal–Wallis test (n.s., non-significant; * *p* < 0.05; ** *p* < 0.01).

Daily Ration Size (% BMi.day^−1^)	K1 (%)		K2 (%)	
	Control	Probiotic	Control	Probiotic
1	−7.87 ± 2.61 ^a^	3.33 ± 3.14 ^b^	-	83.33 ± 45.3
2	16.88 ± 0.75 ^a^	23.33 ± 3.47 ^b^	36.30 ± 1.88 ^a^	49.74 ± 6.68 ^b^
3	13.08 ± 1.61 ^a^	18.53 ± 0.19 ^b^	20.87 ± 2.57 ^a^	27.25 ± 0.27 ^b^
4	11.25 ± 2.85 ^a^	15.09 ± 1.80 ^a^	15.62 ± 3.96 ^a^	19.86 ± 2.37 ^a^
6	7.54 ± 0.45 ^a^	9.73 ± 2.01 ^a^	9.27 ± 0.55 ^a^	11.59 ± 02.40 ^a^
Ration-size effect	*	**	*	*

BMi, initial tank biomass.

**Table 6:** Feed, gross energy and digestible energy intake per diet group at the calculated maintenance and optimal feed ration size. Estimate of the “scope for growth” and predicted growth-rate of shrimps at optimal ration size.

Feed and Growth Indices		Maintenance Ration (Rm)		Optimal Ration (Ropt)	
		Control	Probiotic	Control	Probiotic
Feed intake	(g.kg^−1^.day^−1^)	11.3	9.4	21.3	18.9
GE intake ^(a)^	(kcal.kg^−1^.day^−1^)	50.9	42.3	95.9	85.1
DE intake ^(b)^	(kcal.kg^−1^.day^−1^)	38.1	31.7	71.9	63.8
SFG	(kcal)			33.8	32.1
GR ^(c)^	(g.kg^−1^.day^−1^)			3.3	4.5

GE, gross energy; DE, digestible energy; SFG, scope for growth; GR, growth rate. ^(a)^ based on basal diet GE = 4502 kcal.kg^−1^ (Table 1); ^(b)^ based on basal diet DE = 3376 kcal.kg^−1^ (Table 1); ^(c)^ predicted optimal growth rate at Ropt.

**Table 7:** Statistical significance of diet, ration size and their interaction for each biochemical parameter measured in the (**a**) digestive gland and (**b**) haemolymph (2-way ANOVA; n.s., non-significant; * *p* < 0.05, ** *p* < 0.01, *** *p* < 0.001).

Parameter		Diet	Ration Size	Diet × Ration Size
(**a**) Digestive Gland				
α-amylase activity	(U.mg_prot_^−1^)	***	***	n.s.
Trypsine activity	(U.mg_prot_^−1^)	**	n.s.	*
α-amylase/trypsine		n.s.	n.s.	n.s.
Glucose	(mg.g_organ_^−1^)	n.s.	**	n.s.
Glycogene	(mg.g_organ_^−1^)	**	*	n.s.
TAS	(µmol.g_organ_^−1^)	***	***	n.s.
(**b**) Hemolymph				
Glucose	(mg.mL^−1^)	*	*	n.s.
TAS	(µmol.mL^−1^)	n.s.	*	n.s.

TAS, total antioxidant status.

**Table 8:** Specific activities of digestive enzymes in the digestive gland of *L. stylirostris* per test diets at each ration size tested. Values given as mean ± SD with n = 3. For each parameter, different letters within the same raw indicate significant differences between diets by pairwise comparisons using Fisher’s Protected Least Significant Difference (PLSD). The ration-size effects were assessed by a Kruskal–Wallis test (n.s., non-significant; ** *p* < 0.01; *** *p* < 0.001).

Daily Ration Size (% BMi.day^−1^)	α-amylase Activity (U.mg_prot_^−1^)		Trypsin Activity (U.mg_prot_^−1^)		α-amylase/Trypsin	
	Control	Probiotic	Control	Probiotic	Control	Probiotic
1	4.03 ± 0.12 ^a^	5.77 ± 0.29 ^b^	0.38 ± 0.04 ^a^	0.33 ± 0.04 ^a^	11.96 ± 1.16 ^a^	18.50 ± 2.26 ^b^
2	4.56 ± 0.27 ^a^	5.52 ± 0.11 ^b^	0.30 ± 0.03 ^a^	0.38 ± 0.05 ^a^	16.00 ± 1.80 ^a^	16.29 ± 0.73 ^a^
3	3.62 ± 0.26 ^a^	4.43 ± 0.20 ^b^	0.31 ± 0.03 ^a^	0.27 ± 0.02 ^a^	11.99 ± 2.47 ^a^	16.91 ± 1.45 ^a^
4	2.63 ± 0.54 ^a^	3.05 ± 0.12 ^a^	0.22 ± 0.04 ^a^	0.23 ± 0.01 ^a^	12.69 ± 1.36 ^a^	13.40 ± 0.66 ^a^
6	3.19 ± 0.31 ^a^	3.90 ± 0.31 ^a^	0.18 ± 0.02 ^a^	0.32 ± 0.06 ^b^	17.80 ± 2.61 ^a^	15.60 ± 4.63 ^a^
Ration size effect	***	**	**	n.s.	n.s.	n.s.

BMi, initial tank biomass.

## Data Availability

The data presented in this study are available on request from the corresponding author.
